# Quantitative assessment of cleft volume and evaluation of cleft’s impact on adjacent anatomical structures using CBCT imaging

**DOI:** 10.1007/s11282-023-00736-0

**Published:** 2024-02-01

**Authors:** António Vicente, Anna-Paulina Wiedel, Magnus Becker, Susanne Brogårdh-Roth, Xie-Qi Shi, Kristina Hellén-Halme

**Affiliations:** 1 Department of Oral and Maxillofacial Radiology, Faculty of Odontology, Malmö University, Carl Gustafs Väg 34, 214 21 Malmö, Sweden; 2 Department of Oral and Maxillofacial Surgery, Skåne University Hospital, Malmö, Sweden; 3 Department of Clinical Sciences in Malmö, Lund University, Malmö, Sweden; 4 Department of Plastic and Reconstructive Surgery, Skåne University Hospital, Malmö, Sweden; 5 Department of Pediatric Dentistry, Faculty of Odontology, Malmö University, Malmö, Sweden; 6 Section of Oral and Maxillofacial Radiology, Department of Clinical Dentistry, University of Bergen, Bergen, Norway

**Keywords:** Orofacial cleft, Cone-beam computed tomography, Cleft volume, Alveolar bone grafting

## Abstract

**Objectives:**

To determine pre-operative cleft volume and evaluate cleft´s impact on surrounding anatomical structures in children and adolescents with orofacial clefts using cone bean computed tomography (CBCT) imaging.

**Methods:**

The present retrospective study retrieved CBCT examinations of 68 patients from a previous study. The examinations had been exposed either before (*n* = 53) or after (*n* = 15) alveolar bone grafting. Pre-operative volume of cleft was determined, and type and location were evaluated. Morphological changes on the adjacent anatomical structures, including the incisive foramen, the nasal septum and floor, and the inferior turbinate, were assessed.

**Results:**

Mean bilateral cleft volume was 0.76 cm^3^, while mean unilateral cleft volume was 1.08 cm^3^; the difference was significant (*p* < 0.001). Variation in cleft volume, however, was large. The incisive foramen was not visible in the majority of cases with bilateral clefts (71%); the difference was significant (*p* = 0.001). In cases with unilateral clefts, the nasal septum in 87% was curved towards the cleft or graft side. Also, the mean size of the widest part of the inferior turbinate was 8.8 mm on the cleft or graft side and 10.4 mm on the non-cleft side. The difference was significant (*p* < 0.001).

**Conclusions:**

When required, CBCT is a feasible method for quantitatively illustrating alveolar clefts and their impact on the morphological development of surrounding structures. Variation in cleft volume was large.

## Introduction

Cleft lip and/or palate (CL/P) defects are the most frequent congenital abnormalities found in the craniofacial area [1]. According to a recent systematic review [2], global prevalence is 0.45 per 1000 live births. Orofacial clefts are caused by a developmental disturbance of the maxilla and palate in the first three months of gestation and have a multifactorial etiology. They can be isolated non-syndromic clefts, or a part of a syndrome [3]. The condition has physical, aesthetic, psychologic, and emotional consequences. It can affect speech and cause malocclusion, as well as become a reason for mockery in school [4]. The classification of these malformations has three main categories: cleft of the lip only, cleft of the palate only, and cleft of both lip and palate [4]. A multidisciplinary team of specialists is required for management [1], which is a long-term process [5] with the main goal of re-establishing feeding and speech capacities as well as aesthetics [6]. Alveolar bone grafting is a surgical procedure performed when the patient reaches mixed dentition [5], between 9 and 12 years of age [7]. Autogenous bone grafts are used to repair the clefts of the maxillary alveolar process with the main goal being to bond the segments of the maxilla to close the oronasal fistula [7]. The outcome of the bone transplant must be appraised before orthodontic treatment is begun [5].

Ionizing radiation presents a higher risk to children and adolescents than adults. The tissues of children and adolescents replicate at a faster rate and are thus more vulnerable to DNA damage. Furthermore, children and adolescents have a longer post-exposure life expectancy than adults, thus providing more time for tumors to develop [8]. Consequently, when deciding the necessity of radiation exposure to children and young persons, a strict consideration about the need in every case should be applied [9, 10]. Radiographic imaging plays an important role at different phases during the treatment process in children and adolescents with orofacial clefts for different diagnostic questions. Oenning A. et al. list important clinical indications in CL/P cases: location, shape, size, and volume of the defect; eruption control of adjacent teeth; nasal cavity involvement; and treatment plans for bone grafts and orthognathic surgery [10]. After treatment, radiographic imaging is used to monitor healing, follow up tooth eruption, and plan subsequent treatment of residual clefts [10]. Cone-beam computed tomography (CBCT) produces multiplanar cross-sectional and 3D reconstructions [8]. The effective dose of CBCT is lower than of computed tomography (CT), but still much higher than traditional dental radiographs [8]. In fact, a recent study has reported that CBCT could be responsible for cytotoxic and genotoxic effects on buccal mucosa cells in children and adolescents. Thus, CBCT cannot be considered a risk-free examination [11]. According to SEDENTEXCT (safety and efficacy of a new and emerging dental X-ray modality) guidelines, CBCT is preferred over multi-slice computed tomography (MSCT) for cleft assessment, with the smallest necessary volume size selected [12]. Nevertheless, like any other radiographic modality, CBCT should never be a routine examination [8]. Use of ionizing radiation should always follow the principle of ALADAIP (as low as diagnostically acceptable being indication-oriented and patient-specific) [13]. A recent study demonstrated how an ultra-low-dose CBCT protocol provided sufficient image quality in the radiographic evaluation of children and young persons with alveolar clefts, both before and after alveolar bone grafting. This was a radiation dose reduction of approximately 70% when compared to the standard-dose CBCT protocol. Structure visibility was similar in the two protocols [14]. Regardless, and when necessary, the 3D evaluation that CBCT provides is a clear advantage over other radiographic methods in cases of CL/P [15]. CBCT is able to portray thickness and height of the alveolar bone, particularly on the buccal and palatal sides. Conventional, 2D dental radiography cannot provide such information [16]. Furthermore, the previous studies have demonstrated how pre-surgical knowledge of the exact volume of bone graft needed can improve surgical results [17–19]. Complications, such as postoperative pain, nerve injury, and pelvic instability due to the pelvis being the graft donor site, increase the risk of donor site morbidity [19]. According to de Rezende Barbosa et al., using 3D imaging to evaluate the entire cleft and determine its dimensions is more accurate than any other method [17]. Thus, several ways of calculating cleft volume by CBCT examination have been presented [19]. However, a few clinical studies have assessed cleft volume and evaluated surrounding vital anatomical landmarks in a large group of participants.

The aim of this study was to use CBCT imaging to determine pre-operative cleft volume and evaluate the impact on surrounding anatomical structures in children and adolescents with uni- and bilateral orofacial clefts. Our hypotheses were that there is a large variation in cleft volume and that clefts influence the morphological development of adjacent anatomical structures.

## Materials and methods

### Ethical considerations

The present study retrieved CBCT examinations from a 2016/2017 study [14] that had received ethical approval by the Swedish Ethical Review Authority (Daybook no. [Dnr] 2016/422-31, 2019-04106) [20]. Thus, participants received no ionizing radiation in the present study. The Swedish Ethical Review Authority approved the present study (Dnr 2023-01170-01) [20], which was conducted according to the principles of the Declaration of Helsinki, and the guidelines of the International Commission on Radiological Protection (ICRP) in Biomedical Research [21]. Karolinska Institutet in Stockholm, Sweden, anonymized all CBCT images before sending them to our research group at the Faculty of Odontology in Malmö, Sweden. The only information we received was the sex and age (in years) of the patients at the time of the examination.

### Study design and participants

CBCT examinations of 68 pediatric patients (age range 7–15 years) with non-syndromic clefts involving the alveolar process were carried out in 2016–2017 at Karolinska Institutet in Stockholm, Sweden. One CBCT examination was made for each patient, either before or after alveolar bone grafting. The purpose was to evaluate the anatomy of the anterior maxilla, before or after alveolar bone graft surgery [14]. Fifty-three CBCT examinations were exposed prior to bone graft surgery and 15, after bone graft surgery using a Promax 3D Mid scanner (Planmeca Oy, Helsinki, Finland). Thirty-five volumes were exposed with a standard-dose protocol and 33, with an ultra-low-dose protocol. Both were protocols defined by the manufacturer. The standard-dose protocol had a tube current of either 6 or 8 mA, depending on the size of the patient, and an exposure time of 12 s. The ultra-low-dose protocol had a tube current of either 4 or 5 mA, depending on the size of the patient, and an exposure time of 4 s. Both protocols had a tube voltage of 90 kV. All CBCT images had the same voxel size of 0.2 mm^3^ and a field of view of 8.0 × 5.0 cm. The dose area products were 482 and 612 mGy cm^2^, respectively, for the tube currents of 6 and 8 mA in the standard-dose protocol, and 114 and 141 mGy cm^2^, respectively, for the tube currents of 4 and 5 mA in the ultra-low-dose protocol.

### CBCT examination review

One junior oral and maxillofacial radiologist (AV) with 5 years of experience in oral radiology evaluated all CBCT examinations (*n* = 68) under dimmed-light conditions. Reviews of the first ten cases were done under the supervision of two senior oral and maxillofacial radiologists (X-QS, KHH), each with more than 10 years of experience in oral radiology. In cases of doubt or disagreement, consensus was reached within the group (AV, X-QS, and KH-H). The technical settings of all CBCT examinations were extracted with ImageJ software (ImageJ, version 1.53q, US National Institutes of Health, Bethesda, Maryland, USA). Tube voltage, exposure time, tube current, dose area product, and field of view as well as voxel size were noted.

**Fig. 1:**
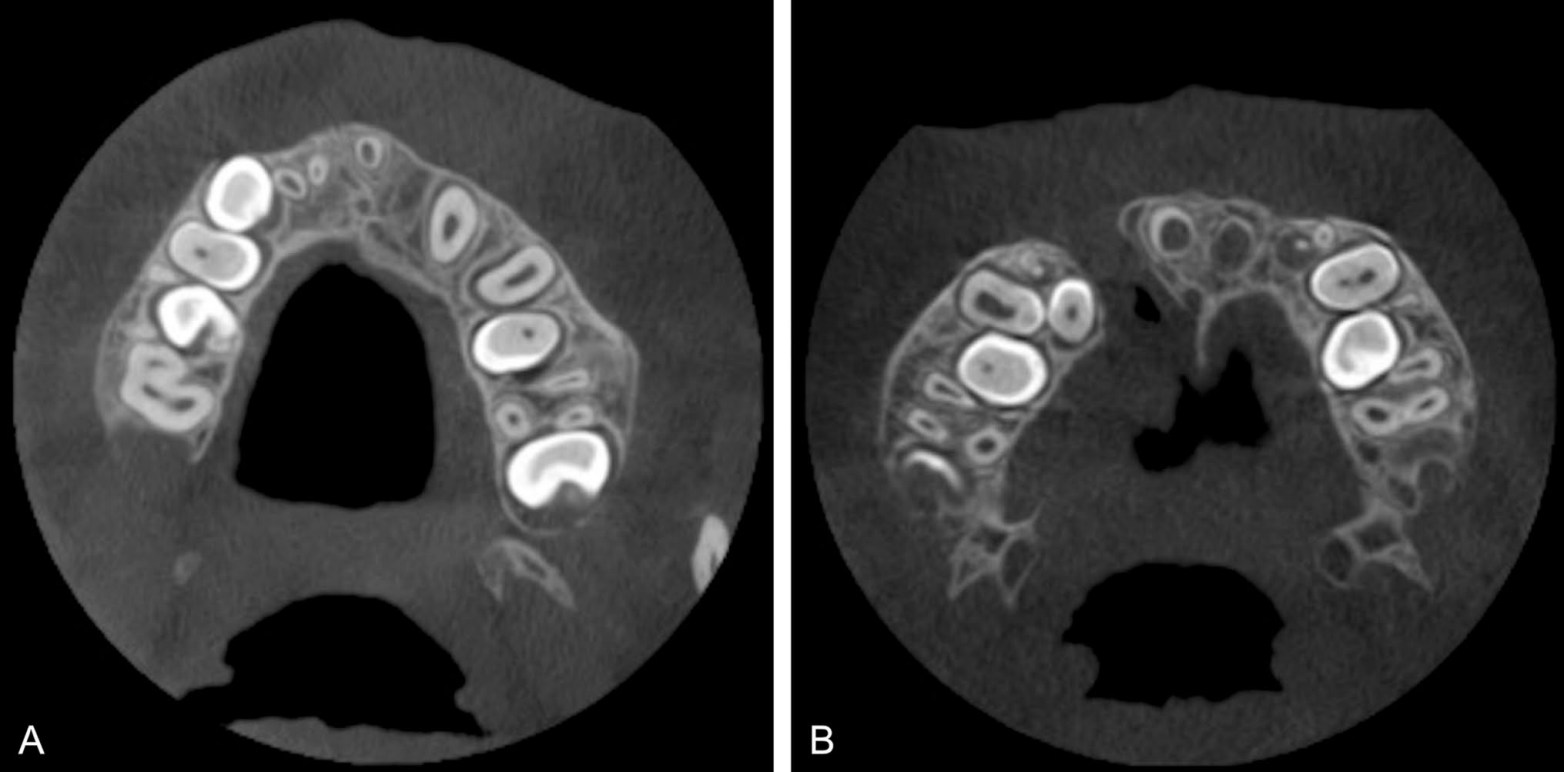
Visibility of the incisive foramen: **A** completely visible, **B** partially visible

**Fig. 2:**
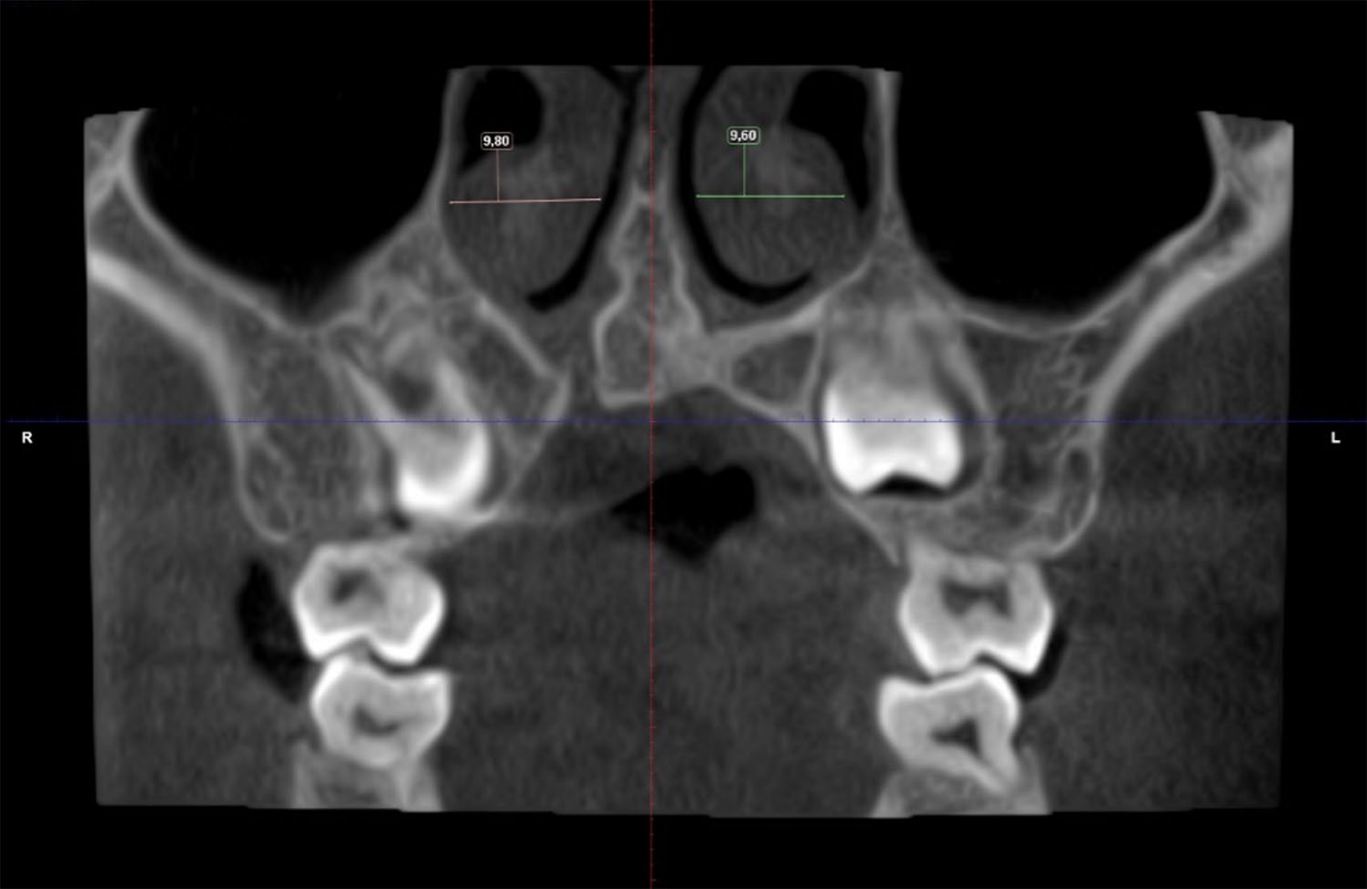
Example of determining the size of the widest part of the inferior turbinate

**Fig. 3:**
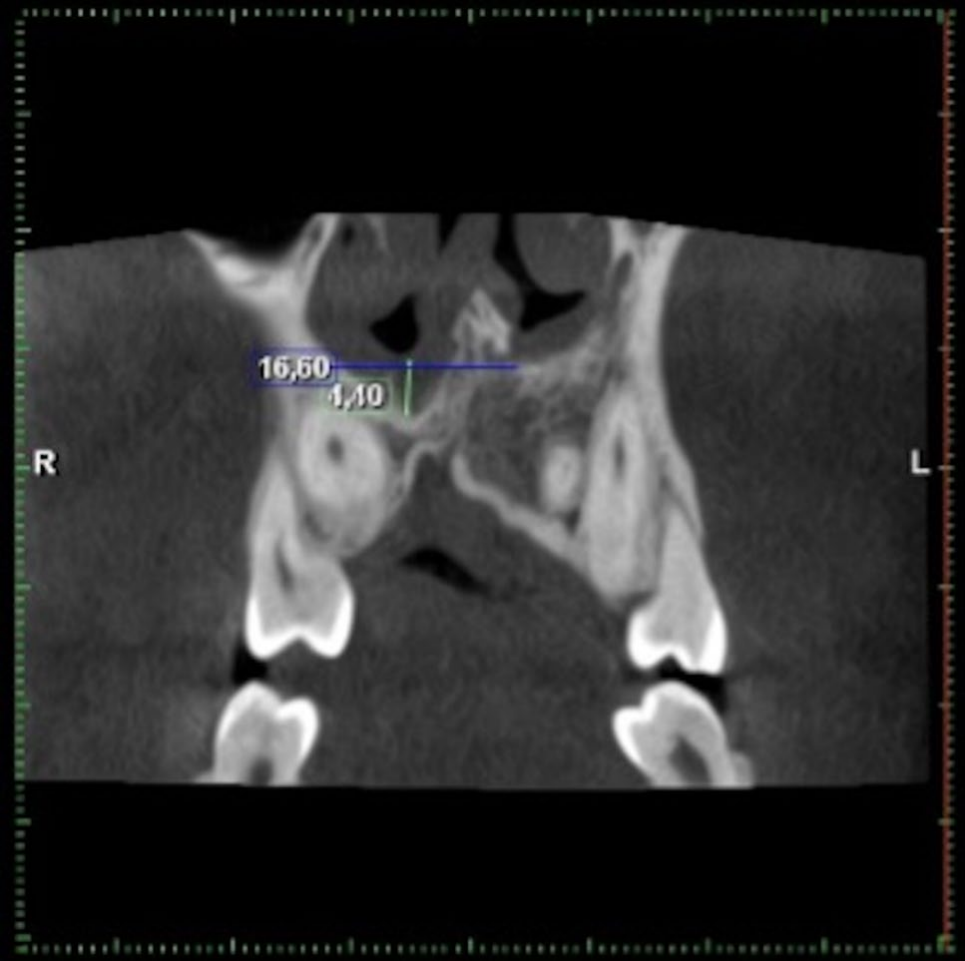
Comparison of the location of the nasal floor between the graft side (right side) and the non-cleft side (left side)

Table 1 summarizes the parameters evaluated in the CBCT examinations and Figs. 1, 2 and 3 present examples. All parameters, apart from cleft volume, were evaluated using Planmeca Romexis software (Romexis, version 6.0, Helsinki, Finland).

**Table 1:** Parameters collected from cone beam computed tomography (CBCT) examinations of 68 pediatric patients with cleft lip and/or palate

Parameters derived from the CBCT examinations
Pre-operative volume of the cleft*
Type of cleft^#^
Unilateral
Bilateral
Location of the cleft^#^
Right side
Left side
Both sides
Visibility of the incisive foramen^#^ (Fig. 1)
Not visible: no cortical border was visible
Partially visible: only part of the cortical border was visible
Completely visible: foramen with well-defined cortical border
Nasal septum deviation (evaluated in the anterior part of the nasal cavity)^#^
Deviation to the cleft side: curve towards cleft/graft side
Deviation to the non-cleft side: curve towards non-cleft side
No deviation: straight nasal septum
Size of the widest part of the inferior turbinate on the cleft or graft side in relation to the non-cleft side in unilateral clefts^#^ (Fig. 2)
Width in mm of the widest part of the inferior turbinate, measured in the coronal view
Post-operative location of the nasal floor on graft side in relation to the non-cleft side^#^ (Fig. 3)
Nasal floor on the graft side, superior to the non-cleft side
Nasal floor on the graft side, inferior to the non-cleft side

*Evaluated using the Bruker CTAn Micro-CT software (Bruker CTAn Micro-CT, version 1.15.4, Billerica, MA, USA)

^#^Evaluated using the Planmeca Romexis software (Romexis, version 6.0, Helsinki, Finland)

### Volume determination

Cleft volume in the 53 pre-operative CBCT examinations was determined using Bruker CTAn Micro-CT software (Bruker CTAn Micro-CT, version 1.15.4, Billerica, MA, USA). The examinations were imported in DICOM format.

**Fig. 4:**
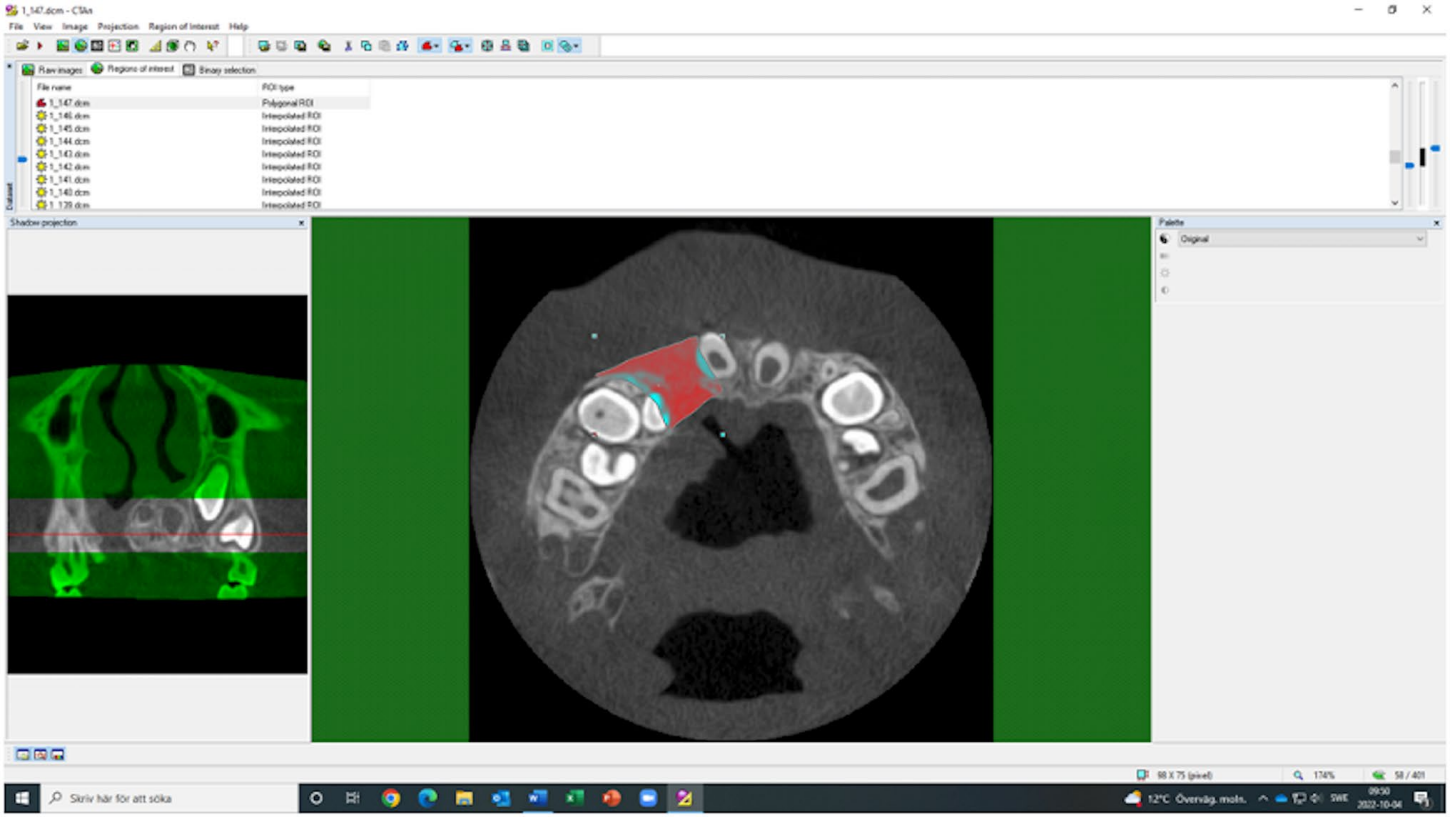
Definition of the cleft area (in red)

The anatomical reference point for the superior border of the cleft was defined as the nasal floor of the non-cleft side in cases with unilateral clefts and as the expected nasal floor in cases with bilateral clefts. The inferior border was defined as the marginal part of the alveolar crest, where the enamel cement junction of adjacent teeth was visible. The buccal, palatal, mesial, and distal borders of the cleft were roughly defined manually on all axial cross-sectional images (Fig. 4).

The software calculated the volume of the cleft, taking account of the limits imposed by the user. These included the volume within the borders of the cleft as described anteriorly and excluded structures and tissues that should not be considered part of the cleft, such as tooth substance. Volume was calculated in cubic pixels and then converted into cubic centimeters.

### Statistical analysis

All data were registered and analyzed in the Statistical Package for the Social Sciences (IBM SPSS Statistics for Windows, version 27.0. Armonk, NY: IBM Corp). Frequency analyses were performed, and cross-tabulations were analyzed. The statistical tests used included the *t* test for comparison of cleft volumes and the mean size of the inferior turbinates, and the ordinal regression analysis for evaluating the visibility of the incisive foramen. The significance level was set at *p* ≤ 0.05.

## Results

Fifty-four of the cleft cases were unilateral clefts, of which 40 were examined with CBCT before, and 14 after alveolar bone graft surgery. Fourteen cases were bilateral clefts, of which 13 were examined with CBCT before, and 1 after alveolar bone graft surgery.

### Pre-operative cleft volume

Mean unilateral cleft volume was significantly larger than bilateral cleft volume (*p* < 0.001; Table 2).

**Table 2:** Cleft volume calculated from cone beam computed tomography examinations of 53 pediatric patients with cleft lip and/or palate before alveolar bone graft surgery

Type of cleft	*n*	Cleft volume		
		Minimum (cm^3^)	Maximum (cm^3^)	Mean ± SD (cm^3^)
Unilateral	40	0.19	2.01	1.08 ± 0.47
Bilateral	13	0.13	1.51	0.76 ± 0.37

*SD* Standard deviation

Variation in cleft volume, however, was large in all cases, both uni- and bilateral.

### Morphological characteristics of the anatomical structures surrounding the cleft

The incisive foramen was not visible in the majority of bilateral cleft cases (71%). The results of the ordinal regression analysis showed a significant association between the cleft type, i.e., uni- and bilateral cleft, and the likelihood of visibility of incisive foramen. The visibility of the incisive foramen in the unilateral cleft group was nine times more likely to be in a higher category on the visibility scale compared to those with bilateral clefts (95% confidence interval, *p* = 0.001).

In 87% of the cases with a unilateral cleft, the nasal septum deviated towards the cleft or graft side. In only 7.4% did it deviate towards the non-cleft side and in 5.6%, was straight. Also, in cases of a unilateral cleft, the mean size of the widest part of the inferior turbinate was 8.8 mm on the cleft or graft side and 10.4 mm on the non-cleft side. The difference was significant (*p* < 0.001). Specifically, 14 CBCT examinations of cases with a unilateral cleft were made after alveolar bone grafting. In 10 cases, the nasal floor on the graft side had an inferior position in relation to the nasal floor on the non-cleft side. In two cases, the nasal floor on the graft side had a superior position, and in two cases, the position was neutral.

## Discussion

The present study found that CBCT appears to be feasible for volumetric assessment of alveolar clefts and their impact on anatomical landmarks in the region and supported our hypothesis of large variations in cleft volume. The same software used to assess volume is also able to produce a 3D model of the clefts, which can supplement pre-surgical planning and aid in explaining treatment for the patient and legal guardians. These are further uses of pre-operative CBCT examinations, if indicated. Previous studies have found that diagnostic information on alveolar cleft volume prior to alveolar bone grafting seems to improve surgical outcomes [17, 19]. An incorrect amount of graft material can cause various problems, such as graft failure or undue resorption [18]. The systematic review of Kapila and Nervina concluded that CBCT allows accurate assessment of cleft volume, which is optimal for planning alveolar bone graft surgery, by determining the precise amount of bone necessary [23]. The present study calculated pre-operative cleft volume using the CTAn software with CBCT examinations exposed in a clinical setting. A previous study successfully tested different volumetric assessment methods, although in plastic phantoms [17]. Mean unilateral cleft volume (1.08 cm^3^) was significantly higher than mean bilateral cleft volume (0.76 cm^3^; *p* < 0.001). The amount of space available for each cleft in a bilateral cleft case may explain our finding. However, different orthodontic treatment approaches can also impact cleft volume due to expansion of the maxilla. Furthermore, the large variation in cleft volume that we found has clear implications for the pre-surgical planning of alveolar bone grafting with suitable radiographic modality, that is in most cases 2D radiographs. The present study also used the retrieved CBCT examinations to analyze different anatomical landmarks in the region, which can be useful for clinicians. Our hypothesis here was that clefts influence the morphological development of adjacent anatomical structures. The systematic review of De Grauwe et al. considered CBCT to be excellent for monitoring the development of teeth adjacent to the cleft area and root morphology as well as evaluating the outcome of alveolar bone graft surgery. All of the above can be achieved in a CBCT exam with a lower radiation dose and better image quality than CT [24]. The incisive canal is an anatomical structure located anteriorly in the hard palate, connecting the oral and nasal cavities. It makes space for the nasopalatine nerve and the sphenopalatine artery [22]. The incisive foramen is the buccal opening of the incisive canal [25]. Both identification and evaluation of the incisive canal before surgical interventions in the area are important for their success, and prevention of complications [25]. Thus, the incisive foramen is an important anatomical landmark for alveolar bone graft surgery. It is important for surgeons to know the exact location of this nerval opening. In the present study, the incisive foramen was not visible in the majority of cases with bilateral clefts, and the difference was significant. Caution is recommended in these cases. The cleft can in fact involve or be situated in the incisive canal [25].

Closing the cleft lip and palate negatively affects growth, and subsequently, the morphology of the nose in terms of a deviated septum and hypertrophic nasal turbinate [25]. That could be observed in the present study. A deviated septum has been known to affect normal respiration [26]. Deviation and deformity of the nasal septum are usually present in patients with CL/P [27], and some nasal airway problems will persist, even after surgical treatment [28]. Moreover, the nasal obstruction and mouth-breathing resulting from a deviated septum can cause malocclusion [29, 30]. Septum deviation can also be the reason for esthetic problems in young patients with CL/P [29]. In the present study, the nasal septum in the majority of cases with unilateral clefts was curved towards the cleft or graft side, which is in line with the previous studies [27, 29, 31]. Using CBCT, the Jiang et al.’s study reported that the degree of nasal septum deviation was significantly related to the severity of the cleft [29]. Deviation of the septum to the cleft side causes narrowing of the nasal cavity on that side, and as a result of the concavity, widening on the non-cleft side [31]. Furthermore, evaluating nasal asymmetry is important in the treatment of patients with CL/P, to improve function and aesthetics [29]. The turbinates or conchae are major anatomical structures located within the walls of the nose. Their functions include respiration, filtration, and humidification. Conditions like deviation of the nasal septum can cause obstruction of the nose and affect turbinate functioning [26]. Inferior turbinate hypertrophy (ITH) occurs in patients with CL/P defects. ITH is particularly troublesome, since it causes a reduction in healthy nasal area, and leads to nasal obstruction on both sides. Development of the maxillofacial skeleton may become disturbed [32]. The present study has shown that the mean size of the widest part of the inferior turbinate was greater on the non-cleft side when compared to the cleft or graft side, which agrees with other studies [32, 33]. ITH usually develops on the opposite side of the deviated septum due to increased airflow through the unobstructed nostril [34], which would explain our finding. Pinto et al.’s study reported finding a substantial degree of ITH and nasal airway dysfunction in patients with unilateral CL/P [32]. The present study has shown that the position of the nasal floor on the graft side of 71% of the cases with unilateral clefts was inferior to the nasal floor on the non-cleft side. Such pre-surgical information should be useful for the plastic surgeon who is planning secondary corrections of the nose.

CBCT is, when required, a useful radiographic method in cleft cases. However, for the post-surgical control, usually made 6 months after alveolar bone grafting, a recent study has shown that CBCT is not required as a complement to the 2D radiograph, if graft failure can be observed clinically, or if the 2D radiograph shows an open residual cleft [35].

### Study limitations

The main limitation of the present study was the lack of a gold standard. We could only assume the estimated volume of the patient’s cleft. However, the same problem exists in the clinical setting.

### Study strengths

The present study was clinical and included CBCT examinations of patients in a normal clinical situation, in contrast to laboratory studies made on phantoms. Furthermore, the number of CBCT examinations was relatively high and their quality had been confirmed in a previous study [14].

### Clinical relevance

The findings of this study can provide further evidence on the clinical uses of CBCT imaging before and after alveolar bone grafting in patients with orofacial clefts. Knowledge of cleft shape and volume and of the morphology of adjacent anatomical structures can facilitate therapeutic planning prior to alveolar bone grafting, and orthodontic or orthognathic treatment.

## Conclusions

When required, CBCT is a feasible method for quantitatively illustrating alveolar clefts and their impact on the morphological development of surrounding structures. Variation in cleft volume was large.

## Data Availability

The datasets used and analysed in the present study are available from the corresponding author upon reasonable request.
